# Cardiac Tamponade as a Complication of Microscopic Polyangiitis: A Case Associated With a COVID-19 mRNA Vaccine

**DOI:** 10.7759/cureus.37569

**Published:** 2023-04-14

**Authors:** Cesar Avalos, Yasaman Ahmadzadeh, Dmytro Gatsak, Syed Ahmad Moosa, Mohammad Ali Mozaffari, Alexander S Imas, Regina Miller

**Affiliations:** 1 Rheumatology, State University of New York Downstate Medical Center, New York, USA; 2 Internal Medicine, Roger Williams Medical Center, Providence, USA; 3 Internal Medicine, State University of New York Downstate Medical Center, New York, USA; 4 Internal Medicine, St. John's Episcopal Hospital, Far Rockaway, USA; 5 Research, Bangladesh Medical Association of North America, New York, USA; 6 Internal Medicine, Corewell Health, East, Dearborn, USA; 7 Internal Medicine, Mount Sinai Hospital, New York, USA; 8 Internal Medicine, Kings County Hospital Center, Brooklyn, USA

**Keywords:** mrna vaccine, coronavirus disease 2019 (covid-19), anca vasculitis, systemic vasculitis, microscopic polyangiitis

## Abstract

Widespread uptake of the coronavirus disease 2019 (COVID-19) vaccinations has become the world’s championed defense against the global pandemic. Four vaccines have been either approved or authorized for emergency use by the FDA, and at this time, over 13 billion doses of these vaccines have been administered around the world. Unfortunately, uncommon and sometimes unforeseen side effects such as small-vessel vasculitis have been reported. In this case report, we present a 74-year-old woman with a history of hypertension, type 2 diabetes mellitus, and hypothyroidism who developed microscopic polyangiitis (MPA) following the second dose of the Pfizer-BioNTech mRNA vaccine for COVID-19. The diagnosis of MPA was confirmed by a kidney biopsy. The autoimmune condition progressed to pericardial effusion and eventual cardiac tamponade, which is occasionally seen in the disease. In this patient’s case, we suspect there to be a temporal association between mRNA COVID-19 vaccination and the development of MPA. Direct causation has not been determined.

## Introduction

With over 13 billion doses already administered, the coronavirus disease 2019 (COVID-19) vaccination drive is one of history's most extensive immunization campaigns [1]. All four vaccines (Pfizer-BioNTech, Moderna, Johnson & Johnson’s Janssen, and Novavax) that are either approved or authorized for emergency use in the United States have been known to be safe and effective in preventing severe disease and/or hospitalization.

As vaccination rates increase, rare side effects begin to emerge. Cases of small-vessel vasculitis have been reported following the administration of the different COVID-19 vaccines [2-10]. Only a few instances of microscopic polyangiitis (MPA) following Pfizer-BioNTech mRNA vaccine administration have been reported in English literature [2-6]. MPA is a systemic necrotizing small-vessel vasculitis that predominantly affects the pulmonary and renal systems. This article reports a case of MPA that developed after the second dose of the Pfizer vaccine. The hospital course of our patient was further complicated by an associated pericardial effusion - a rare complication of MPA - progressing to cardiac tamponade.

## Case presentation

A 74-year-old female with a history of hypertension, type 2 diabetes, and hypothyroidism was sent by her primary care physician to the emergency department (ED) with the presentation of malaise, fatigue, chest pain, and a lower extremity rash described as non-tender, erythematous, and macular. The patient had received Pfizer-BioNTech’s mRNA vaccine for COVID-19 two weeks earlier. Before her ED visit, outpatient laboratory work was performed, and the significant findings are shown in Table 1.

**Table 1:** Abnormal outpatient laboratory results of the patient led to her ED visit. *HPF: high power field.

Variable	Outpatient laboratory and imaging results	Reference range if applicable
Abnormal blood tests		
Erythrocyte sedimentation rate (mm/h)	73	0–20
C-reactive protein (mg/dL)	5.3	<1
Blood urea nitrogen/BUN (mg/dL)	50	8.0–23.0
Serum creatinine (mg/dL)	3.78	0.50–0.90 (patient’s baseline: 0.90-1.1)
Abnormal urinalysis findings		
Urine protein	Positive (2+ = 100 mg/dL)	Negative
Urine RBCs	25–50/HPF*	0–5/HPF

Additionally, outpatient transthoracic echocardiography was unremarkable (with left ventricular ejection fraction >50% and no significant valvular pathology); renal Doppler revealed no evidence of renal artery stenosis or obstructive uropathy. Lab work conducted 53 days before the patient’s second dose of the COVID-19 vaccine was normal, including a negative urinalysis and BUN/Cr within normal ranges. She had received her first dose of the vaccine approximately seven months earlier.

After her outpatient work-up, the patient was referred to the ED, complaining of generalized weakness, malaise, fatigue, and pleuritic chest pain, which had progressively worsened after her second dose of Pfizer-BioNTech’s COVID-19 mRNA vaccine. On admission, the lower extremity exam was significant for 3+ edema. On skin exam, an erythematous, nonpainful, diffuse macular rash was noted. Inpatient laboratory testing supported the outpatient findings, including a BUN of 62 mg/dL and a creatinine level of 4.78 mg/dL; a urinalysis positive for proteinuria at 100 mg/dL; urinary sediment of RBC 50-100/HPF (reference range 0-5/HPF); and a urine protein to creatinine ratio of 4.117 (reference range 21-161). A transthoracic echocardiogram on admission demonstrated a normal left ventricular ejection fraction of 50-55% and an additional small pericardial effusion.

**Figure 1:**
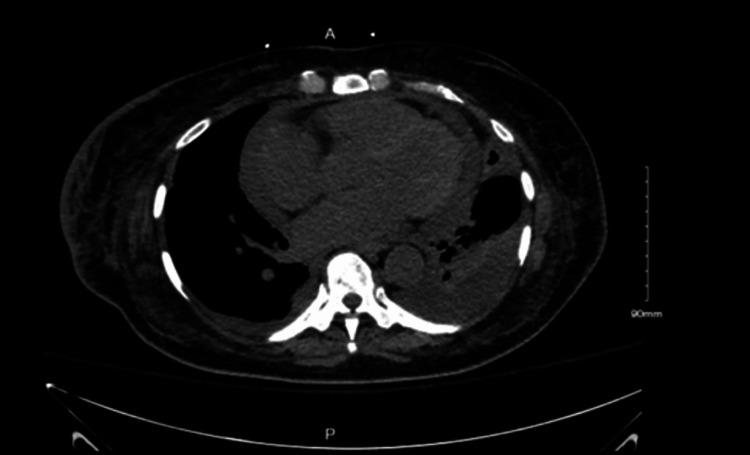
Moderate-to-large pericardial effusion on CT chest.

A high-resolution chest computed tomography (CT) was performed to assess lung involvement, and the patient was found to have a moderate-to-large pericardial effusion (Figure 1). Follow-up transthoracic echocardiography confirmed a moderate pericardial effusion with a reduction in left ventricular ejection fraction compared to the prior study (LVEF 40-45%) and now a new-onset right ventricular dilated cavity with reduced right ventricular systolic function consistent with cardiac tamponade. The patient then underwent emergent pericardiocentesis, with 350 cc of serosanguinous fluid drained.

**Figure 2:**
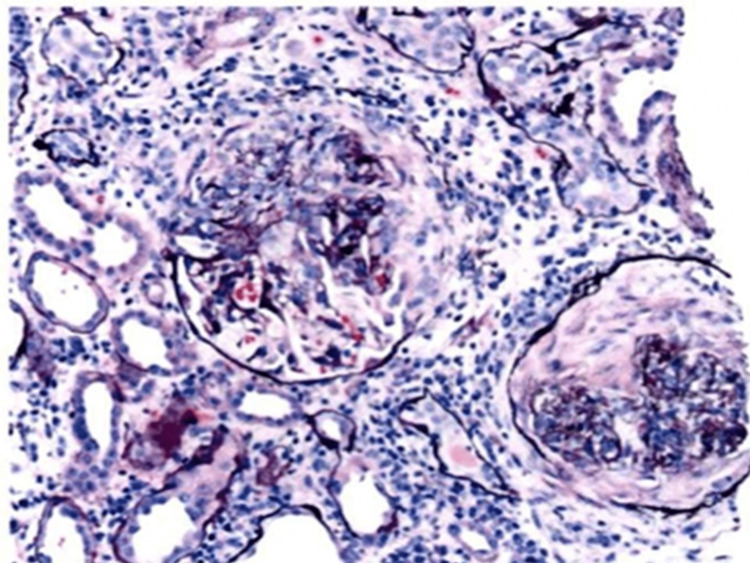
Light microscopy evaluation of renal tissue shows evidence of crescents, a classical histopathological lesion in severe forms of rapidly progressive glomerulonephritis.

Given the patient’s constellation of systemic symptoms in conjunction with renal dysfunction without secondary causes and progressively worsening pericardial effusion, antineutrophil cytoplasmic antibodies (ANCA) were sent, which was noted to be positive for P-ANCA at a ratio of 1:160 (reference range 1:20). Other autoimmune and infectious serologies were unremarkable, including antinuclear antibody (ANA), rheumatoid factor (RF), anti-citrullinated protein antibody, complement level, HIV, rapid plasma reagin (RPR), and hepatitis B and C. On renal biopsy, she was found to have MPO-ANCA-mediated crescentic focal necrotizing and focal sclerosing glomerulonephritis (Figure 2).

The patient was subsequently started on 1 g of intravenous methylprednisolone for five days, followed by oral prednisone at 1 mg/kg. She further received two doses of rituximab 1-g infusions, 14 days apart, given the underlying MPO-ANCA-associated vasculitis. For the pericardial effusion, she was transferred to a facility specializing in pericardial window surgery.

## Discussion

Vaccines remain the lynchpin of our response to the COVID-19 pandemic. Pfizer-BioNTech’s mRNA vaccine is currently approved by the United States Food and Drug Administration (FDA) for use as a two-dose primary series for preventing COVID-19 in individuals 12 years of age and older [11,12]. It has emergency-use authorization (EUA) for a third dose in this population as a booster. For selected younger populations (six months to fourteen years old), the FDA has also issued an EUA for primary and booster doses.

As the immune system strengthens in response to various vaccines, mild symptoms (including fever, fatigue, headache, and local reactions) are expected. Before the availability of COVID-19 vaccines, the question of whether influenza vaccines could cause autoimmune disease remained debatable [13]. One study proposed molecular mimicry and immune cross-reactivity as potential reasons for vaccine-induced autoimmunity [14]. Molecular mimicry occurs when there is considerable similarity between certain components within the vaccine and specific human proteins. This similarity may result in immunological cross-reactivity, a phenomenon where the immune system's reaction towards the pathogenic antigens may cause harm to similar human proteins. Examples of vaccines suspected to be associated with such immunological cross-reactions include those used against influenza (H1N1), hepatitis B virus (HBV), and human papillomavirus (HPV). Unfortunately, high-quality scientific data regarding these adverse events is lacking. There is also a dispute concerning the relationship between ANCA vasculitis and influenza vaccination [15]. Although an association between the Pfizer vaccine and MPA may exist, a causal relationship remains unclear.

Alternatively, a report by Jeff et al. suggested that RNA-based vaccination increased ANCA production [16]. These authors also observed the reduction of the ANCA response to the RNA vaccine once it was treated with ribonuclease. ANCA vasculitis has been reported in connection with COVID-19 infection and the different types of vaccination to prevent it [2-6,17,18], prompting the suspicion of whether the immunological phenomenon is a direct result of the mRNA content of the vaccine. Hypothesized mechanisms like molecular mimicry, defective neutrophilic apoptosis, polyclonal activation, and a systemic proinflammatory cytokine response can explain the temporal causal association between autoimmune manifestations of ANCA vasculitis and COVID-19 vaccines. In all the reported cases, management involved steroids in combination with rituximab or other immunomodulating medications (e.g., azathioprine).

Although uncommon, about 10% of MPA cases develop pericarditis [19], and there have been a few reports of cardiac tamponade secondary to this autoimmune disease [20].

We propose that the mRNA vaccine potentially triggered this patient’s MPA, eventually leading to cardiac tamponade. Her blood work before receiving her second dose of the Pfizer vaccine was considered unremarkable, and derangements in renal lab work afterward prompted concern about the proposed association. Post-marketing surveillance and reporting remain essential with the widespread utilization of mRNA vaccines.

## Conclusions

Although an association between the mRNA COVID-19 vaccines and the development of vasculitis seems evident, causation remains to be established. However, clinicians should have a low threshold for diagnosing vasculitis secondary to such vaccinations, particularly ANCA-associated vasculitis such as MPA.

A timely diagnosis of MPA is critical given the high mortality rate (~90%) if left untreated in the first year after diagnosis. Although MPA typically involves the pulmonary and renal organs, a careful lookout should be kept for other organ involvement, especially cardiac, given the immediate need for intervention if tamponade physiology is present.
